# Case report:Multiple abscesses caused by *Porphyromonas gingivalis* diagnosed by metagenomic next-generation sequencing

**DOI:** 10.3389/fmed.2022.1089863

**Published:** 2023-01-26

**Authors:** Yichen Zhang, Youfeng Zhu, Huijuan Wan

**Affiliations:** ^1^Department of Intensive Care Unit, Guangzhou Red Cross Hospital, Guangzhou, China; ^2^Department of Otorhinolaryngology Head and Neck Surgery, The First Affiliated Hospital, Sun Yat-sen University, Guangzhou, China

**Keywords:** *Porphyromonas gingivalis*, next generation sequencing (NGS), multiple abscesses, metronidazole, percutaneous catheter drainage

## Abstract

**Background:**

Extraoral infection by *Porphyromonas gingivalis* (*P. gingivalis*) is extremely rare and challenging to diagnose because the fastidious pathogen is difficult to culture by traditional methods. We report the first case of a patient with multiple abscesses in muscles and the brain with dura empyema due to *P. gingivalis*, which was diagnosed by metagenomic next-generation sequencing (mNGS).

**Case presentation:**

A 65-year-old male patient was admitted to our hospital for multiple lumps in his body. Brain magnetic resonance imaging (MRI) and lower-limb computed tomography (CT) revealed multiple abscesses in the brain and muscles. A diagnosis of *P. gingivalis* infection was made based on mNGS tests of blood, cerebrospinal fluid (CSF), and pus samples, as the traditional bacterial culture of these samples showed negative results. Target antibiotic therapy with meropenem and metronidazole was administered, and CT-guided percutaneous catheter drainage of abscesses in both thighs was performed. The size of muscle abscesses reduced significantly and neurological function improved. The patient was followed up for 4 months. No abscesses re-appeared, and the remaining abscesses in his backside and both legs were completely absorbed. He can speak fluently and walk around freely without any neurological deficits.

**Conclusion:**

Metagenomic next-generation sequencing is helpful for early diagnosis and subsequent treatment of *P. gingivalis*-associated multiple abscesses.

## Introduction

*Porphyromonas gingivalis* (*P. gingivalis*) is a highly adapted non-motile gram-negative, and oral anaerobe. The bacterium is strongly associated with periodontitis, which can destroy the tissues supporting the tooth and eventually lead to tooth loss (1). It can produce various virulence factors such as proteolytic enzymes, capsules, fimbriae, and lipopolysaccharides that can cause tissue destruction, severe inflammation, and sometimes abscess formation (2). Apart from oral infection, *P. gingivalis* can also cause extraoral infections (3–9), such as brain abscesses, chest wall abscesses, subdural empyema, and gas gangrene.

The diagnosis of *P. gingivalis* using conventional methods, such as blood, cerebrospinal fluid, or pus culture is difficult and time-consuming. *P. gingivalis* is a fastidious pathogen that is difficult to culture and requires samples to be transported to the appropriate laboratory immediately under strict anaerobic conditions. The culture time of *P. gingivalis* is approximately 7–10 days, which can lead to delayed diagnosis and therefore inappropriate treatment. Currently, metagenomic next-generation sequencing (mNGS) is being used to identify pathogens with high sensitivity and specificity; it can also lead to an etiological diagnosis in comparatively lesser time (10). Herein, we present the first case of multiple abscesses in a patient’s muscles and the brain with dura empyema due to *P. gingivalis*, which was diagnosed by mNGS.

## Case presentation

A 65-year-old male patient was admitted to our hospital for multiple abscesses. Two weeks before admission to our hospital, the patient presented with an abscess in his left palm. Four days later, three more bumps were found on his right and left thighs and his backside with mild distending pain. Five days prior to admission, as his palm abscess enlarged, he presented to the outpatient department of our hospital. He was treated with levofloxacin (100 mg, intravenous [iv], qd), and his palm abscess was removed by surgical drainage. However, his symptom worsened quickly. He developed fever, headache, gait instability, and dysarthria. The bumps in his thighs and his backside became larger, with severe distending pain in these places.

**FIGURE 1:**
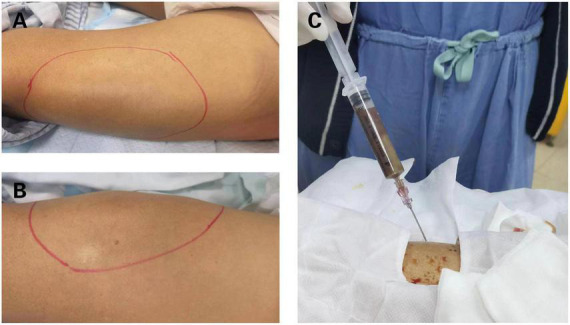
Clinical manifestation of bilateral thighs abscesses. **(A)** Bulging mass on the right thigh. **(B)** Bulging mass on the left thigh. **(C)** Brown fetid pus was aspirated by computed tomography (CT)-guided percutaneous puncture in right thigh abscess.

On admission, the initial physical examination showed a body temperature of 38.5°C, respiratory rate of 19/min, pulse rate of 85/min, and blood pressure of 120/73 mmHg. The cardiovascular and respiratory systems were normal. Abdomen examination did not show tenderness or hepatosplenomegaly. In addition, bumps were found on his left thigh (21 cm × 13 cm) (Figure 1A), right thigh (22 cm × 12 cm) (Figure 1B), and backside (7 cm × 3 cm) with tenderness and fluctuation. The local skin temperature was higher than that of the surrounding skin. Motor weakness of the left upper limb (muscle strength grade 4) and right lower limb (muscle strength grade 2) with bilateral hypotonicity of the upper extremities was observed. There were no signs of hyperreflexia or meningeal irritation. Both sides of the Babinski sign were negative. The patient denied any disease that can influence the immune system, such as tumors, acquired immunodeficiency syndrome (AIDS), hematological diseases, autoimmune diseases, or diabetes mellitus. He also denied the administration of any immunosuppressive agents or steroid drugs. His lymphocyte count was 1.31 × 10^9^ cells/L, the percentage of CD3+ and CD4+ lymphocytes was 31% (normal range 27–51%), and the percentage of CD3+ and CD8+ lymphocytes was 35% (normal range 15–44%). The presence of antibodies of the human immunodeficiency virus was also negative.

**FIGURE 2:**
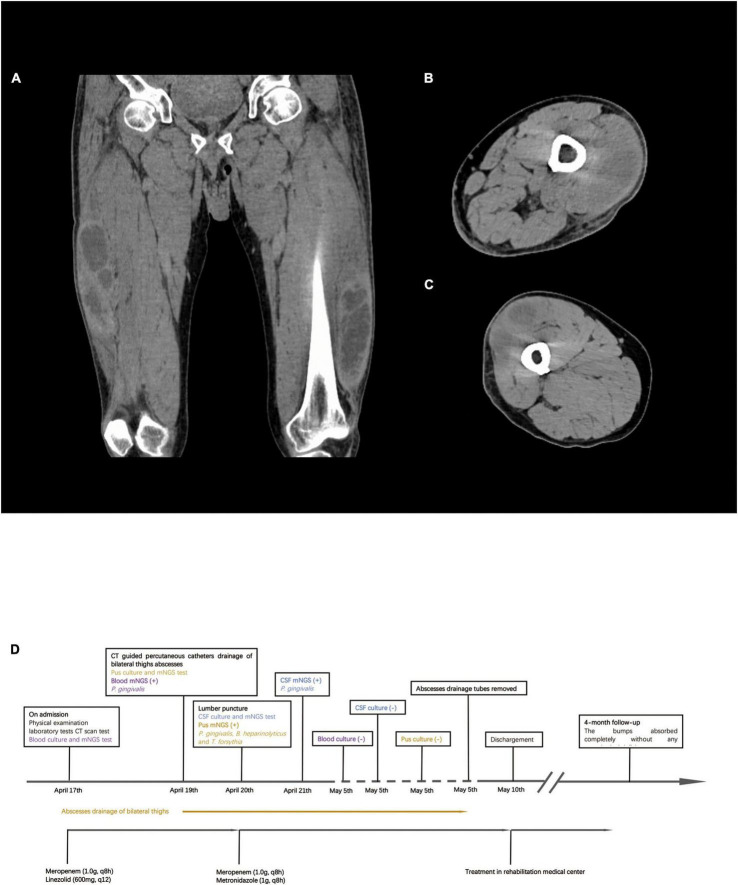
Computed tomography (CT) scan result of bilateral lower extremities and timeline of treatment. **(A)** CT scan shows multiple muscle abscesses in bilateral thighs. **(B)** Right thigh abscess. **(C)** Left thigh abscess. **(D)** Timeline of treatment.

**FIGURE 3:**
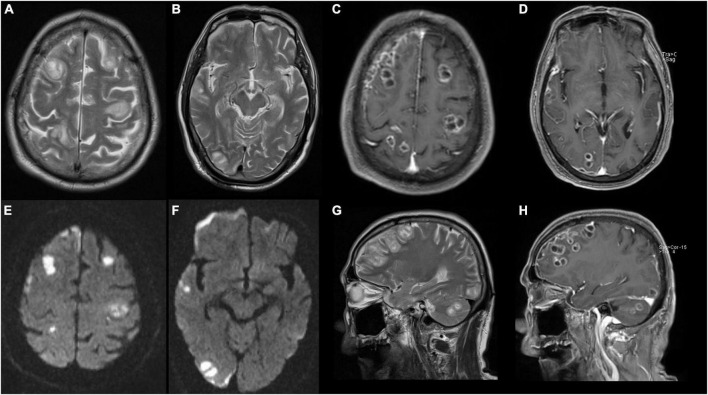
Magnetic resonance image (MRI) of brain shows abscesses in bilateral frontal lobe, right temporo-parieto-occipital lobe, and right cerebellar hemisphere with ring contrast enhancement and perilesional edema. **(A,B,G)** T2-weighted imaging. **(C,D,H)** T2 contrast-enhancement imaging. **(E,F)** Diffusion-weighted MRI.

Computed tomography (CT) of both lower extremities showed the presence of multiple hypodense lesions, which indicated multiple muscle abscesses in the right vastus intermedius, left vastus lateralis muscle, and left adductor magnus (Figures 2A–C). Brain magnetic resonance imaging (MRI) showed rim-enhancing lesions with perilesional edema that indicated bilateral brain abscesses in the frontoparietal lobe, right temporal occipital lobe, and right cerebellar hemisphere (Figure 3). Brain MRI also showed heterogeneously thickened dura with peripheral rim enhancement, which indicated dura empyema on the right frontoparietal lobe (Figure 3). Diffusion-weighted MRI shows a hyperintense signal within these lesions (Figure 3). A lumbar puncture was performed, which showed an intracranial pressure of >300 mmH_2_O (normal range: 80–180 mmH_2_O). The cerebrospinal fluid (CSF) was clear and colorless. The CSF analysis showed the following results: leukocyte count, 99,000 cells/mL; glucose levels, 3.6 mmol/L (normal range: 2.5–4.5 mmol/L); and protein levels, 1,113.9 mg/L (normal range 150–450 mg/L). Based on these findings, a diagnosis of multiple abscesses in muscles and the brain with dura empyema was made, and empirical antibiotic treatment of meropenem (1.0 g, iv drip, q8h) and linezolid (600 mg, iv drip, q12) was started. Anaerobic and aerobic cultures of blood samples and aerobic culture of CSF samples were both performed. CT-guided percutaneous catheter drainage of abscesses was performed in both thighs, and brown fetid pus was aspirated (Figure 1C). The pus sample was used to find the etiological agent using methods of traditional aerobic culture and mNGS test. Stereotactic puncture drainage of brain abscesses was proposed but not performed because the patient refused any type of intracerebral surgery.

**FIGURE 4:**
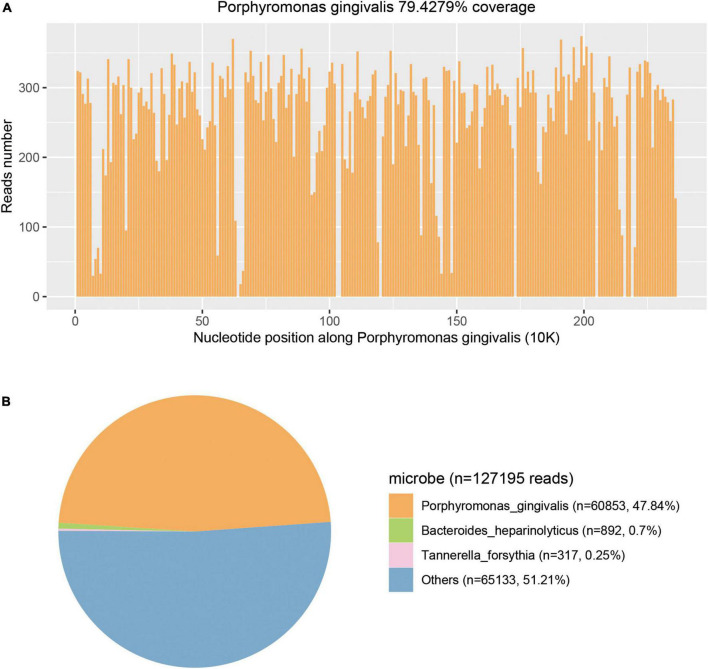
Metagenomic next-generation sequencing (mNGS) results of this case. **(A)** Courage of *Porphyromonas gingivalis* detected by mNGS was 79.4279%. **(B)** A total of 60853 specific reads of *P. gingivalis* were detected by mNGS in this case.

PACEseq mNGS test (Hugobiotech, Beijing, China) using a pus sample from the right thigh abscess was performed to identify the causative pathogens. A QIAamp DNA Micro Kit (QIAGEN, Hilden, Germany) was used for DNA extraction, and a library of total DNA was built with QIAseqTM Ultralow Input Library Kit for Illumina (QIAGEN, Hilden, Germany). Qubit (Thermo Fisher Scientific, MA, USA) and Agilent 2100 Bioanalyzer (Agilent Technologies, Santa Clara, CA, USA) were used to access the quality of the DNA library. The qualified library was finally sequenced on a Nextseq 550 platform (Illumina, San Diego, CA, USA). After sequencing, adapters and short, low-quality, and low-complexity reads were removed from the raw data. Human DNA was also filtered out by mapping to the human reference database. The remaining reads were finally aligned to the Microbial Genome Database.^1^ *Porphyromonas gingivalis* (60,853 specific reads), *Bacteroides heparinolyticus* (892 specific reads), and *Tannerella forsythia* (317 specific reads) were detected on day 3 (Figure 4). Blood and CSF samples were also used to detect pathogens using the method of the PMseq mNGS test (BGI-Shenzhen, Shenzhen, China). *Porphyromonas gingivalis* was detected both in the blood (489 specific reads) and CSF (587 specific reads) samples on day 2 and day 4 as negative results were obtained with the traditional culture of these samples. The blood, pus, and CSF samples were incubated in BACTECTM FX BC systems (Becton Dickinson, Heidelberg, Germany) for 5 days, but no positive signal appeared. As a result, the anaerobic and aerobic cultures of the blood sample and the aerobic cultures of pus and CSF samples were negative.

As *P. gingivalis* was detected, the antibiotic regimen was changed to meropenem (1.0 g, iv drip, q8h) and metronidazole (1 g, iv drip, q8h) on day 3. Chest and abdominal CT and cardiac ultrasonography were performed to identify the source of infection, but no positive result was found in either examination. A dental examination was performed, which revealed moderate oral hygiene with loss of all teeth and chronic gingivitis. We did not find any periodontal abscess. The antibiotic regimen of meropenem and metronidazole was continued until he was discharged from the ICU. The pigtail catheters were left in both legs to allow for continuous drainage with daily flushing by metronidazole. On day 9, the drainage volume was <10 ml of serous-like liquid, and the patient’s white blood cell count was back to normal levels. CT of both legs revealed a remarkable reduction in the size of the abscesses in the right vastus intermedius, left vastus lateralis muscle, and left adductor magnus. The drainage tube was removed on day 18. The size of the bump in his backside was reduced to 3 cm × 2 cm. His neurological function was improved partially. He could speak fluently, but the muscle strength of his right lower limb was still weak (muscle strength: grade 2). As his symptoms improved and his vital signs stabilized, he was discharged from the ICU on day 23 to a rehabilitation medical center. The patient was followed up for 4 months. No abscesses re-appeared, and the remaining abscesses in his backside and both legs were completely absorbed. His muscle strength improved significantly. He returned to normal health. At the time of generating this report, the patient could speak and walk around freely and had no evident neurological deficits. The timeline of the treatment is shown in Figure 2D.

## Discussion

*Porphyromonas gingivalis* is a gram-negative oral anaerobe and is considered a major pathogenic agent causing periodontitis. However, *P. gingivalis* can be disseminated from the oral cavity to other body sites and cause extraoral infections, such as brain abscess, subdural empyema, chest wall abscesses, otitis media, appendicitis, and gas gangrene (3–9). Brain abscesses caused by *P. gingivalis* have been reported in several studies (3–6). Recently, Tanaka et al. (8) reported the first case of chest subcutaneous abscess caused by *P. gingivalis*. To the best of our knowledge, muscle abscesses and brain abscesses in the same patient caused by *P. gingivalis* have not been reported. Here, we reported the first case of multiple abscesses in muscles and the brain with dura empyema caused by *P. gingivalis*.

The diagnosis of brain abscess by *P. gingivalis* mainly depended on bacterial culture because of the lack of characteristic imaging and clinical features. However, the positive rate of culture for *P. gingivalis* is very low. In our case, traditional bacterial cultures of blood, CSF, and pus samples all revealed negative results. Indeed, many oral bacteria, such as *P. gingivalis*, are fastidious pathogens that are difficult to culture and must be transported to the appropriate laboratory immediately under strict anaerobic conditions. Moreover, it takes weeks or more to identify pathogens by traditional culture. Currently, mNGS can simultaneously detect various types of pathogens such as bacteria, viruses, fungi, and parasites in virtually any body fluid type, ranging from low-cellularity spinal fluid to purulent fluids, with high sensitivity and specificity and in far less time than traditional culture tests (10). In our case, *P. gingivalis* was detected within 2 days by mNGS, much earlier than by traditional culture tests, which detected no pathogens, given that the samples were sent to the laboratory at the same time. This proves that mNGS can be more beneficial than traditional testing methods in identifying potential pathogens such as *P. gingivalis*.

In a pus sample from the right thigh, 60,853 (47.84%) specific sequences of *P. gingivalis* were detected in the mNGS test. Other sequences (*n* = 65,133, 51.21%) included non-specific microbial sequences (*n* = 64,713, 50.88%) that did not indicate any specific microbe and relatively fewer environmental bacterial sequences (*n* = 420, 0.33%). Sequences specific for *P. gingivalis* accounted for 97.39% of all specific sequences detected by mNGS. Thus, *P. gingivalis* was the major microbe identified in the pus sample. The non-specific microbial sequences could be unspecified genome regions of *P. gingivalis* and the minor accompanying flora as no specific sequences of other species were found. As specific sequences of *P. gingivalis* were also detected in blood and CSF samples, we confirmed that *P. gingivalis* was the pathogenic bacterial species of multiple abscesses.

In our case, percutaneous puncture of bilateral femoral abscesses was performed and brown fetid pus was aspirated. Unfortunately, only aerobic culture of the pus sample was performed. As *P. gingivalis* is an anaerobic species that cannot live with oxygen, the results of aerobic cultures of pus and CSF samples were negative. However, we did confirm that there were few if any aerobes in these samples. Tanaka et al. (8) reported a case of empyema necessitans that presented as a subcutaneous chest wall abscess caused by *P. gingivalis*. In this case, percutaneous puncture of the subcutaneous abscess was performed, and foul-smelling chocolate-colored pus was aspirated. Gram smears of the pus revealed gram-negative rods, but aerobic culture showed a negative result, similar to our case. Indeed, foul-smelling pus always suggests an anaerobic infection; thus, anaerobic culture is needed.

Some previous studies have also reported brain abscesses caused by *P. gingivalis*. In these cases, patients either had a chronic odontogenic disease or had recently undergone dental surgery before brain abscesses were found. As no other sources of infection were found, they concluded that *P. gingivalis* was derived from the oral.

Targeted antibiotic therapy of meropenem and metronidazole was administrated after pathogens were detected. *Porphyromonas* spp. shows high susceptibility to penicillin, amoxicillin, amoxicillin–clavulanate, metronidazole, tetracycline, and clindamycin (11). In our case, metronidazole was also used to flush the abscess cavities *via* pigtail catheters. The size of muscle abscesses declined significantly, and neurological function improved. Some case reports have revealed that early absorption of brain abscesses leads to a good neurological prognosis (3, 5, 12, 13). In our case, stereotactic puncture drainage of brain abscesses was proposed by our neurosurgical colleagues, but the patient did not agree to the procedure. This may be why the neurological deficit did not improve as significantly as the size of muscle abscesses before discharge.

In our case, the patient denied any diseases that can decrease immune function and denied the administration of any immunosuppressive agents or steroid drugs. His lymphocyte counts and CD4+ and CD8+ percentages were in the normal range. As a result, the immune function of the patient was accessed as normal. In most case reports concerning abscesses caused by *P. gingivalis*, the patients’ immunologic functions were normal, which indicates that anyone can be infected by this microbe. The patient in our case ultimately recovered fully. No abscesses re-appeared, and the remaining abscesses were all completely absorbed without any neurological deficits. The mortality rate and morbidity rate of extraoral *P. gingivalis* infections have never been reported. However, most case reports indicate that with proper treatment, the patient should experience a good recovery.

## Conclusion

We report here the first case of multiple abscesses in muscles and the brain with dura empyema caused by *P. gingivalis*. The mNGS test is helpful in the detection and identification of these pathogens, especially when traditional culture tests show negative results. With antibiotic therapy of metronidazole and meropenem combined with percutaneous catheter drainage of abscesses in both thighs, the size of the muscle abscesses declined significantly, and neurological function improved.

## Data availability statement

The datasets presented in this article are not readily available to protect patient confidentiality and privacy. Requests to access the datasets should be directed to the corresponding author.

## Ethics statement

The studies involving human participants were reviewed and approved by the Ethics Committee of Guangzhou Red Cross Hospital. The patients/participants provided their written informed consent to participate in this study. Written informed consent was obtained from the individual(s) for the publication of any potentially identifiable images or data included in this article.

## Author contributions

All authors listed have made a substantial, direct, and intellectual contribution to the work, and approved it for publication.

## Footnotes

1
https://www.ncbi.nlm.nih.gov/genome
